# Towards the Development of a More Accurate Monitoring Procedure for Invertebrate Populations, in the Presence of an Unknown Spatial Pattern of Population Distribution in the Field

**DOI:** 10.3390/insects9010029

**Published:** 2018-02-27

**Authors:** Natalia B. Petrovskaya, Emily Forbes, Sergei V. Petrovskii, Keith F. A. Walters

**Affiliations:** 1 School of Mathematics, University of Birmingham, Birmingham B15 2TT, UK; 2 Harper Adams University, Newport, Shropshire TF10 8NB, UK; eforbes@harper-adams.ac.uk (E.F.); keith.walters@imperial.ac.uk (K.F.A.W.); 3 Department of Mathematics, University of Leicester, Leicester LE1 7RH, UK; sp237@le.ac.uk

**Keywords:** population size, spatial density distribution, sampling plan, sampling grid, coarse grid, sparse data, bootstrapping

## Abstract

Studies addressing many ecological problems require accurate evaluation of the total population size. In this paper, we revisit a sampling procedure used for the evaluation of the abundance of an invertebrate population from assessment data collected on a spatial grid of sampling locations. We first discuss how insufficient information about the spatial population density obtained on a coarse sampling grid may affect the accuracy of an evaluation of total population size. Such information deficit in field data can arise because of inadequate spatial resolution of the population distribution (spatially variable population density) when coarse grids are used, which is especially true when a strongly heterogeneous spatial population density is sampled. We then argue that the average trap count (the quantity routinely used to quantify abundance), if obtained from a sampling grid that is too coarse, is a random variable because of the uncertainty in sampling spatial data. Finally, we show that a probabilistic approach similar to bootstrapping techniques can be an efficient tool to quantify the uncertainty in the evaluation procedure in the presence of a spatial pattern reflecting a patchy distribution of invertebrates within the sampling grid.

## 1. Introduction

Understanding the spatiotemporal ecology of invertebrates is important for developing strategies to enhance biodiversity and encourage species of conservation interest and strategies for efficient pest management [1]. Assessment of the population size or the average population density of invertebrate species in ecosystems often provides a basis for decision-making in both nature conservation and integrated pest management (IPM). Such assessments must be sufficiently accurate as an unreliable estimate of the total population size can lead to inadequate decision-making, such as unnecessary application of control measures and associated damage to the ecosystem [2]. Inaccurate evaluation of the total population size (or average density) may also result in a loss of important information about the properties of ecological dynamics. This can lead to incorrect conclusions, for instance, about the existence of a strong correlation (synchronisation) between population fluctuations in different habitats [3] or between different species, e.g., between pest and natural enemy populations [4].

A range of methods have been developed to collect information about species abundance, each addressing the specific biological traits of different organisms, as well as the needs of conservation or crop management advisors [5,6,7,8]. The differing biological characteristics of species found within a single habitat or crop often result in the simultaneous use of different methods both in research programmes or by consultants in their decision-making, e.g., [9,10]. Ensuring that population or distribution estimates are comparable between species is therefore important [4]. Both in research programmes and consultancy, the situation can be complicated by the use of different assessment methods and by uneven (heterogeneous) spatial distribution manifested as patches, each patch containing higher numbers of a defined species than in adjacent areas of the habitat [11,12].

In research programmes a sampling grid of traps is frequently deployed across the monitored area, and a time series of repeated standard samples covering the period of interest are analysed. Trap counts can be used to quantify the population abundance of the monitored species using appropriate indices (e.g., the average trap count), either by calculating the population density by dividing the average trap count by the monitoring area [13,14] or by using more advanced algorithms [15,16]. Abundance estimates can provide important data for conservation decision-making [17], and both approaches are often used. Cost implications often restrict the use of grid sampling in IPM, and other factors such as sample size and distribution of sampling points need to be established by research (which can involve grid sampling) to support accurate assessment by growers and consultants [18,19]. It is therefore important that uncertainty in the assessment of population abundance using coarse or fine sampling grids is quantified and understood.

The accuracy of an estimate of the average population density is known to depend on the properties of a sampling grid [20,21]. The sampling grid must capture sufficient information about the population distribution in order to adequately represent the population abundance. One important consideration in a sampling protocol is the total number of locations from which samples are taken (say *N*). In some cases, this property is derived from theoretical assumptions [19,22], while in many others it is based on expert judgement [23]. Although available resources often prevent application of the technique, pre-sampling can be used to obtain a sample mean and variance from which an estimate of the number of sample units needed to achieve a specified accuracy can be calculated; e.g., see [18,24,25]. The trade-off between the number of sample units needed to achieve optimal accuracy and the number that can be afforded can still, however, result in practitioners using coarse sampling grids in a trapping procedure. A similar problem occurs when sampling is used for pest management decision-making. The number of sample locations in agricultural fields with linear dimensions measured in several hundred meters has, both historically and in recent practice in major UK field crops, rarely exceeded twenty [9,10,26,27,28], and in some cases *N* can be as small as a single location [29]. Such restrictions on sampling protocols may affect the accuracy of the assessed population abundance. Previous work [30,31,32] suggests that standard evaluation procedures are unlikely to provide accurate results when coarse sampling grids are used because of uncertainty about the patterns of spatial distribution of the invertebrates within the sample area, which lead to unreliable estimates of the population abundance.

The amount of information that needs to be collected to estimate population abundance with a required accuracy depends not only on the number of samples but also on the properties of the spatial pattern of population distribution [16,33]. For an extremely uniform distribution, an adequate sampling grid can consist of a small number of traps—ultimately, a single trap (provided the inherent effects of randomness are properly addressed, which may bring additional constraints, e.g., see Section 2.2 in [16].) A population with a strongly heterogeneous distribution should be monitored using a sampling grid with a larger number of traps to allow for the resolution of all features of a complex spatial pattern [19]. Using a coarse grid in this case risks crucial information about the population being missed and population abundance evaluated with substantial errors.

The problem is exacerbated by the fact that population distribution is usually unknown prior to implementation of the sampling/monitoring. Location of a given trap in relation to high or low density patches is random, and if the objective of the assessment is to estimate average population density, the uncertainty arising from the spatial pattern is inherent to the problem. When a coarse sampling grid is employed, because of this uncertainty, the estimate becomes essentially a random variable, which has to be handled using appropriate probabilistic techniques [31,34].

In this paper, field data on three invertebrate species with different biological and behavioural characteristics collected from agro-ecological systems, by quantifying uncertainty in the assessment of population abundance using coarse sampling grids, is used to further develop the approach discussed in [31,34]. The paper is organised as follows. In Section 2 we briefly revisit the problem of evaluation of the average trap count. As an example, we will use field data from a recent study on a slow moving mollusc, the slug *Deroceras reticulatum* (Müller); because slugs are pests [35], we consider the problem of its population size evaluation in the context of IPM. In Section 3, we discuss the concept of uncertainty in sampling data arising as a result of an unknown spatial pattern in the population distribution. We then consider how the uncertainty can be dealt with using a modified bootstrapping approach to quantify the probabilistic characteristics of the average trap count. Our approach is further tested in Section 4 using published field data on the slow moving, free living terrestrial planarian *Arthurdendyus triangulatus* (Dendy). In Section 5, we demonstrate that our approach can be applied to species from a different taxa such as the highly active carabid beetle *Pterostichus melanarius* (Illiger). Since carabids are known to be a natural enemy that can be used in conservation biocontrol of some pests [36], they are generally regarded as a beneficial species; therefore, we consider its monitoring in the context of conservation. Finally, in Section 6, we consider the implications of our findings in case the population abundance changes in time using multiple trap data on *D. reticulatum* collected over a season. Section 7 provides a discussion and conclusions.

## 2. Evaluation of the Average Trap Count on Coarse Sampling Grids: A Case Study of a Grey Field Slug (***Deroceras reticulatum***) Population

Some key aspects of sampling-grid design that affect the estimation of average population density in the presence of a spatial pattern, and which have previously been applied to the monitoring of beetles (*P. melanarius*) [37] and flatworms (*A. triangulatus*) [38], are unlikely to be species-specific and may remain valid for different taxa of invertebrates such as insects, arachnids, molluscs, and annelids. Despite the broad relevance of the approach, important differences occur when it is applied in the context of IPM (where the objective is often to reduce abundance of the monitored species) and conservation (where the density of species of interest should remain sufficiently high). In IPM, a threshold population (or sample) size may be established at which application of a control procedure is justified on economic grounds [39], and it is important that the assessment procedure ascertains whether this threshold is exceeded with the required level of accuracy. In conservation, the objective may be to take action to prevent populations of defined species declining below an established density, so such action thresholds may be used for different purposes. For the purposes of this study and to avoid discussion of the relevance of specific thresholds, nominal ‘management action thresholds’ will be considered to establish the principles investigated.

The grey field slug (*Deroceras reticulatum*) is a pest of a wide range of agricultural and horticultural crops, resulting in significant economic losses in most years [40,41]. The species is slow moving and has two activity peaks in arable fields, between November and December and again between late February and May [35]. The discontinuous distribution of slugs in arable fields results in readily detected patches of higher slug numbers interspersed within areas of lower slug densities irrespective of the size of slug populations [42,43,44].

Sampling of slug populations in commercial winter wheat crops was carried out between autumn 2015 and autumn 2017 in four commercial winter wheat fields [44]. A standard experimental design was established in all fields in both cropping years. Refuge traps had an upturned 18 cm diameter, and plastic plant pot saucers were placed in a regular 10×10 grid with 10 m between the nearest traps. Sampling grids were established at a minimum of 20 m from the nearest field edge. The number of slugs under each refuge trap were recorded after traps were left undisturbed for 14 days.

The trap counts obtained in a given field on a given date are available as a table with *N* entries, where *N* is the number of sampling locations (N=10×10=100). The average trap count can then be evaluated as

(1) $$S\left(N\right)=\frac{1}{N}\sum_{i=1}^{N}C_{i}$$

e.g., , see [45,46], where $C_{i}$ is the trap count in the *i*th refuge trap.

**Figure 1:**
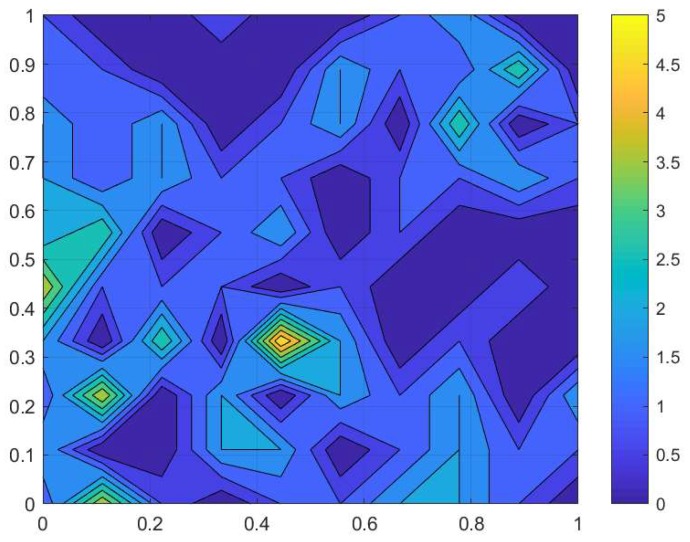
The slug spatial distribution reconstructed from trap counts. Trap counts were taken on a sampling grid of 10×10 locations in the Oadby field on 02 September 2016 (see details in the text), and the corresponding numerical values are given in Figure 2a. The total length *L* of the sampling grid (100 m) in the *x* and *y* directions is rescaled as L=1. The continuous distribution shown in the figure was obtained from the discrete data (see Figure 2a) by using MATLAB software.

**Figure 2:**
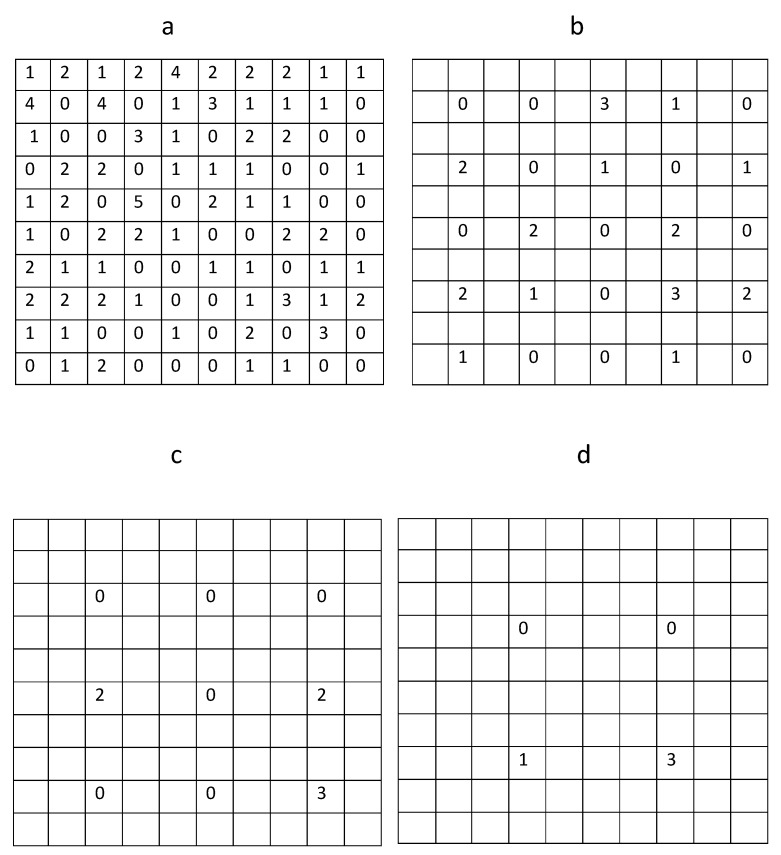
Trap counts on fine and coarse sampling grids. (**a**) Trap counts on the original sampling grid of 10×10 locations. (**b**) Trap counts on a hypothetical sampling grid of 5×5 locations. (**c**) Trap counts on a hypothetical sampling grid of 3×3 locations. (**d**) An extremely coarse sampling grid of 2×2 locations is generated from the original grid.

Our baseline example applies Equation (1) to trap counts collected from a sampling grid in a farm field at Oadby, Leicestershire on 02 September 2016. The slug distribution reconstructed from the data is shown in Figure 1, and the corresponding trap count values are shown in Figure 2a. The average trap count obtained from Equation (1) is low (S=1.07), which is typical for slugs in early autumn.

One important feature of Equation (1) is that the average trap count *S* depends explicitly on the number *N* of sampling locations, S=S(N). In order to demonstrate this, and to investigate whether the result S = 1.07 will significantly change if we use fewer sampling locations, average trap count was re-calculated using a hypothetical trapping grid consisting of every second trap count in each row and each column of the data table when rows and columns were numerated from a reference point in the upper top left corner of the table (see Figure 2b). The average trap count obtained on a coarse grid of 25 sampling locations is $S_{c}=0.88$ (where the subscript ‘*c*’ is used to indicate a coarse grid).

The discontinuous, heterogeneous distribution of the slug population across the sampling grid indicates that reduction in the number *N* of sampling locations may ultimately result in insufficient information about the population being available to obtain an accurate evaluation of the average trap count across the sample area. Thus, optimisation of the number *N* of sampling locations will enable the required level of accuracy to be reached whilst minimising sampling effort.

To compare the average trap count $S_{c}$ with that for *S* the relative error is defined as

(2) $$e=\frac{|S-S_{c}|}{S}.$$

Having substituted the values S=1.07 and $S_{c}=0.88$, we obtain e=0.178, which means that we underestimate the average trap count with the error of approximately 18% when we use a coarse sampling grid of 25 traps instead of our original grid of 100 traps.

A common accuracy requirement used in ecological applications [47] is

(3) e≤τ

where τ is a specified tolerance, i.e., the upper bound for the acceptable values of the estimation error. Since the tolerance up to τ=0.5 is often considered as acceptable [22,47,48], we conclude that removing three quarters of the total number of traps does not make any significant impact on the accuracy of this evaluation of the average trap count.

Generating an even coarser hypothetical sampling grid, by considering every third trap in each row and column of the original table (Figure 2c) and substituting the remaining trap counts into Equation (1) results in the average trap count $S_{c}=0.78$ on a sampling grid of N=9 traps. The error is e=0.273; i.e., we underestimate the average trap count by approximately 27%.

Finally, constructing a very coarse sampling grid of N=4 sampling locations (Figure 2d) by taking every fourth trap count in each row and column of the table in Figure 2a and substituting the four remaining trap counts into (1) gives us $S_{c}=1.0$ and e=0.065. This is a surprising result, as the evaluation of the average trap count obtained when just N=4 sampling locations are used appears to be more accurate than the two previous cases when trap counts at N=25 and N=9 sampling locations were used. In order to resolve this paradox, let us notice that in this choice of sampling locations we started counting rows and columns from the top left corner of the table. Repeating the same evaluation but this time counting every fourth row and fourth column from the top right corner, the average trap count of $S_{c}=1.5$ is evaluated with the error e=0.4019, which appears to be a more logical outcome.

The above result clearly demonstrates that an average trap count estimated on a coarse sampling grid is essentially a random value. In the case where the sampling grid is set up without reference to the location of either slug patches or factors that may influence the distribution of slugs in the field a single estimate is not reliable. However, in cases where the original sampling grid is sufficiently large, it appears possible to provide a reliable estimate by quantifying the statistical properties of the estimate. This will be investigated in the next section.

## 3. Random Selection of Sampling Locations on a Quasi-Regular Grid (***Deroceras reticulatum***)

**Figure 3:**
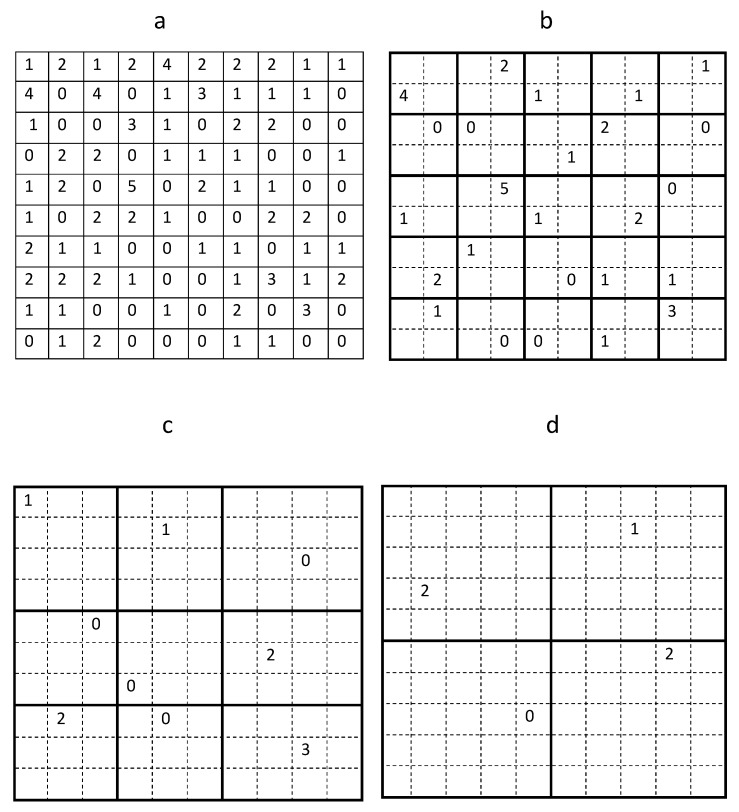
Generating a sequence of quasi-regular coarse sampling grids. Only one trap count is randomly selected with equal probability from each sub-domain on a coarse grid. The boundaries of each sub-domain on a coarse sampling grid are shown as bold lines (see details in the text). (**a**) The original sampling grid of N=100 locations. (**b**) An example of trap counts randomly selected on a quasi-regular sampling grid of N=25 locations. (**c**) A random choice of trap counts on a coarse grid of N=9 locations. (**d**) A very coarse sampling grid where one trap count is randomly selected in each of four sub-domains.

In order to quantify the uncertainty of the average trap count, in this section we generate auxiliary data sets of trap counts using sub-grids of the original sampling grid of data collected in the Oadby field on 02 September 2016. Starting from the original table of trap counts, which corresponds to a regular sampling grid of N=10×10 locations shown in Figure 2a, the original sampling grid was divided into 25 sub-domains each containing 4 trap counts as shown in Figure 3b. A single trap count in each sub-domain was randomly selected resulting in a hypothetical coarse grid of N=25 sampling locations. This ‘quasi-regular’ sampling grid preserves a regular structure whilst allowing the position of each trap being shifted within each sub-domain.

The random choice of a single trap count in each sub-domain has already been discussed in Section 2 but can now be further justified by the following argument. Considering, for example, the top left corner of the original domain. There are four trap counts in the corresponding sub-domain, i.e., {1,2,0,4}; see Figure 3a. A random choice of any of the above values can be interpreted as moving a trap location across the sub-domain. Thus, the location of the sub-domain is fixed; however, depending on the precise trap location, any one of the four values {1,2,0,4} for the trap count might occur. If the distribution of trap counts collected in the field arises from a certain probability density function [49,50,51], then some trap count values are more likely to be recorded than others. However, it is not known from the probability density function where those trap counts can be found in a spatial domain. The same probability density function will generate different spatial patterns at different times. Thus, we can predict the existence of patches with high slug density and patches with low slug density if the probability density function for the slug distribution is available, yet we cannot predict the spatial arrangement of those patches. Hence, the results of the sampling procedure depend not only on the probability density function of the slug population but also on the particular spatial pattern of slug distribution at the time trap counts are collected. Since a priori information about that spatial pattern is usually not available, the optimal location of each trap on a coarse sampling grid cannot be predicted. Therefore, in order to reveal the statistical properties, all possible cases must be simulated.

Using the example of data from the sampling grid of 25 traps shown in Figure 3b, the average trap count is $S_{c}=1.24$ with an error e=0.1589. Clearly this result is different from the answer obtained on the sampling grid with the same number of sampling locations considered in Section 2, which reflects the random nature of the average trap count.

It can be readily seen that, by shifting the location of the trap between the four possible positions within each of the 25 sub-domains, one obtains in total as much as $K=4^{25}$ different sampling grids and, correspondingly, $4^{25}$ possible average trap count values $S_{c}$. Since the average trap count $S_{c}$ is essentially a random variable, this procedure gives us *K* realisations of that random variable. The whole multiplicity of *K* possible values of $S_{c}$ then can be used to establish its statistical properties, e.g., the mean and the standard deviation. This approach is similar to well-known bootstrapping techniques [52].

Each value of $S_{c}$ obtained in the above procedure can be compared with the average trap count *S* obtained from the original (fine) sampling grid of Figure 2a by computing the error (2). For the coarse sampling grid of N=25 locations, it will produce $K=4^{25}$ values of the evaluation error *e*. Since $S_{c}$ is essentially a random variable, *e* is a random variable too. Hence, similarly to $S_{c}$, the whole array of *K* realisations of *e* can be quantified by some statistical measures, e.g., the mean and the standard deviation.

Consider *K* realisations of $S_{c}$ and let the range of $S_{c}$ be $S_{c}\in \left[S_{c}^{min},S_{c}^{max}\right]$. The interval $\left[S_{c}^{min},S_{c}^{max}\right]$ is divided into *M* subintervals or ‘bins’ and the size of each bin is defined as $s=(S_{c}^{max}-S_{c}^{min})/M$. The boundaries of each bin are then defined as $S_{c}^{m+1}=S_{c}^{m}+s$, m=1,2,…,M−1, $S_{c}^{1}=S_{c}^{min}$. The mean value $\mu_{S}$ of $S_{c}$ is given by the following expression:

(4) $$\mu_{S}=\frac{1}{2K}\sum_{m=1}^{M-1}k_{m}\left(S_{c}^{m}+S_{c}^{m+1}\right)$$

where $k_{m}$ is the number of realisations of random variable $S_{c}$ in the subinterval $\left[S_{c}^{m},S_{c}^{m+1}\right]$; for a detailed explanation of Equation (4), see [34].

Equation (4) takes into account that some values of $S_{c}$ are more likely to occur than others. Note that, in general, the mean value of Equation (4) is different from the arithmetic average ${\hat{\mu }}_{S}=\frac{1}{K}\sum_{k=1}^{K}S_{c}^{k}$. The value of $\mu_{S}$ defined by Equation (4) will only coincide with the arithmetic average if estimated on the full ‘fine’ sampling grid, but it may become different when estimated on a coarse grid (i.e., a ‘sub-grid’ of the full grid, cf. Figure 3).

Once the mean $\mu_{S}$ has been computed, the standard deviation is

(5) $$\sigma_{S}=\sqrt{\sum_{m=1}^{M-1}\frac{k_{m}}{K}{\left(\frac{S_{c}^{m}+S_{c}^{m+1}}{2}-\mu_{S}\right)}^{2}}$$

where again we take into account frequencies of finding certain realisations of random variable $S_{c}$.

Similarly, we define the mean value $\mu_{e}$ of error *e* as

(6) $$\mu_{e}=\frac{1}{2K}\sum_{m=1}^{M-1}k_{m}\left(e^{m}+e^{m+1}\right)$$

and the standard deviation as

(7) $$\sigma_{e}=\sqrt{\sum_{m=1}^{M-1}\frac{k_{m}}{K}{\left(\frac{e^{m}+e^{m+1}}{2}-\mu_{e}\right)}^{2}}.$$

Here $k_{m}$ is now the number of realisations of random variable *e* in the subinterval $\left[e^{m},e^{m+1}\right]$, where each sub-interval $\left[e^{m},e^{m+1}\right]$ is defined from the range $\left[e^{min},e^{max}\right]$ in the same manner as has been done for variable $S_{c}$.

The definition of accuracy in Equation (3) now has to be revised to determine that the upper bound of the evaluation error falls below the specified tolerance:

(8) $$\mu_{e}+\sigma_{e}\leq \tau .$$

Consider, for example, the tolerance τ=0.25. If the condition of Equation (8) holds, the coarse sampling grid can be regarded as sufficiently accurate: whatever the geometry of a quasi-regular sampling grid of N=25 sampling locations (i.e., wherever we install a trap in each sub-domain of the grid), we will have an accurate evaluation of the average trap count. The average trap count will be within 25% of the average trap count obtained on the fine grid of N=100 sampling locations. We will further discuss this issue (including the estimation of the probabilities to get an inaccurate answer) in Section 6.

Applying the above procedure to our baseline case of the Oadby field (see Figure 1 and Figure 2a), we can establish whether the condition of Equation (8) holds on sub-grids with a different number *N* of sampling locations. The results of the simulation are shown in Table 1. The second column in the table corresponds to the original grid of N=100 sampling locations. All trap counts contribute equally to Equation (1), and no bootstrapping is possible; thus, only a single average trap count can be computed, i.e., S=1.07. Therefore, both the standard deviation of *S* is zero, and the evaluation error is zero.

The third column in the table corresponds to the range of data sets generated on a quasi-regular grid of N=25 sampling locations. It is not possible to consider all $K=4^{25}$ data sets (sub-grids) as it would require an unrealistically long computation time. Correspondingly, in our statistical experiments, $K=4^{25}$ of all possible realisations of random variables $S_{c}$ and *e* was replaced by a smaller number $K=3\times 10^{5}$. The mean and standard deviation were then computed for $S_{c}$ and *e* using Equations (4)–(7); see the third column of Table 1. The mean value $\mu_{S}=1.092$ of the average trap count is very close to the ‘exact’ answer S=1.07 obtained on the original sampling grid of 100 sampling locations. Furthermore, the standard deviation $\sigma_{c}$ is not large. Hence, for any single trap count selected from four trap counts available in each sub-domain of a coarse sampling grid, a sufficiently accurate estimate of the average trap count is obtained. This conclusion is supported by the associated evaluation error. The mean error is $\mu_{e}\left(N\right)=0.138$, and the upper bound of the error is $\mu_{e}\left(N\right)+\sigma_{e}\left(N\right)=0.238<\tau =0.25$. Thus, for a quasi-regular sampling grid of 25 locations (whichever combination of N=25 trap counts is selected), the evaluation error is unlikely to exceed 25%.

Generating another hypothetical coarse sampling grid that utilises N=9 sampling locations from the original grid, the corresponding domain partition is shown in Figure 3c. This grid preserves a quasi-regular structure so that each of those nine locations can be randomly selected with equal probability from a defined sub-domain of the original grid. Note that, unlike the previous case, it is not possible to include the same number of sampling locations into each sub-domain.

The number of ways to generate a sub-grid using a single trap count from those available in each sub-domain is now $K=9^{4}\times 12^{4}\times 16=2,176,782,336$. Because of the computation time constraints, it is replaced with $K=3\times 10^{5}$. The results are shown in the fourth column of Table 1. It can be readily seen that the evaluation of the average trap count is not sufficiently accurate when a sampling grid of N=9 locations is employed. The mean value of the error remains low, but the larger standard deviation $\sigma_{e}\left(N\right)$ indicates that an accuracy within 25% cannot be reliably achieved using this coarse grid.

Next, a coarse sub-grid consisting of N=4 sampling locations is obtained from the original grid, with the geometry shown in Figure 3d. In this case, a total of $K=25^{4}=390,625$ different data sets (sub-grids) can be generated. The results of the simulation are shown in the fifth column of Table 1. The accuracy of evaluation has decreased as the number *N* of sampling locations is decreased. The mean error is approximately 40%, and the upper error bound is about 70%. This result confirms the conclusion in Section 2 that a sampling grid of four traps will unlikely yield a reliable assessment of average trap count.

Finally, the original sampling grid can be reduced to a ‘grid’ consisting of just one trap. The results are presented in the last column of Table 1. As expected from previous analyses, the accuracy of evaluation is unacceptably low as the mean error is about 80% and the upper error bound is more than 100%.

Once the statistical properties of the average trap count are established, they can be used as a basis for making a management decision. Pest management approaches often recommend an application of pesticides only when the average trap count (being regarded as a proxy of the population abundance) exceeds a defined threshold, say $S_{T}$. The number of traps used to assess population abundance is important, as too few will result in inaccurate decision-making, but the costs associated with assessment can result in prophylactic treatment, an economically attractive option if many traps are used.

In the above, we have shown how the required statistical information about trap count distribution can be obtained: it can be extracted from the data if the grid contains a sufficiently large number of nodes for the bootstrapping procedure described above. In order to make a comparison between this information and the management threshold, one can construct two different quantities that takes into account the range of possible realisations; see $S_{soft}$ and $S_{hard}$ in Table 1. It is these two quantities that should be compared with the threshold. It can be readily seen that one of them provides a stricter test than the other: for instance, on the grid of intermediate coarseness containing 3×3=9 traps, $S_{hard}>S_{T}$ (hence recommending application of control measures), but $S_{soft}<S_{T}$. Which of the two quantities should be chosen in monitoring of a given pest will depend on the acceptable level of risk; in case of a high risk species, a stricter test must be used.

The results of applying the same approach to the evaluation of the average trap count from three different fields where data on slug population were collected in autumn 2016 using a similar sampling grid are presented in Table 2. The slug population in all cases was low (total number of slugs: Adeney Middle field – 58, South Kyme – 112, Uppington – 50). The results of evaluation in Table 2 show the same trend reported for the Oadby field. The mean error along with the upper error bound increase steadily as the number of sampling locations decreases.

As one common property of the field data considered above is a low average trap count, a question may arise here as to whether our approach remains valid in the case of higher counts. This also evokes a question, albeit indirectly, as to how the quality of the estimate may depend on the biological traits of the monitored species. It is well known that the typical degree of spatial aggregation may differ significantly between different species [12]. This may have an effect on the pattern of the population spatial distribution; hence, it may affect the distribution of trap counts. Applying the approach to data collected for other species where the total population is larger may provide some insight into these questions.

## 4. Analysis of Trap Count Data for Flatworm *Arthurdendyus triangulatus*

The New Zealand flatworm, *Arthurdendyus triangulatus* [53], is an invasive free living predaceous terrestrial planarian that is spread by trade or other movement of plants and soil within their country of origin and abroad. It was first reported in the UK in Northern Ireland in 1963 and subsequently in Scotland in 1965, with some established colonies reported in Northern England [54]. Once established in an area, populations can expand into other suitable habitats in search of prey [55,56]. *A. triangulatus* is slow moving and probably feeds exclusively on earthworms [57,58], capturing living prey by wrapping itself around and entangling the worm in a mucus that may play a role in the immobilisation and digestion of the prey [59,60]. Initial work investigating the distribution of flatworms in grassland has included an evaluation of techniques used for trap catch analysis [38] using data previously collected in extensive field studies.

**Figure 4:**
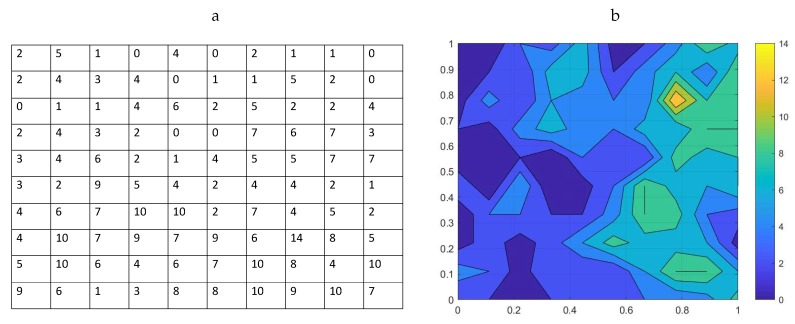
(**a**) An example of trap counts collected by Murchie and Harrison in their field study of flatworm populations; see [61] for more details. (**b**) A flatworm spatial distribution reconstructed (using MATLAB software) from the field data in Figure 4a, where, for convenience, the area’s linear size is scaled to one.

The current study uses data originally collected in January–March of 2002 from a grassland field near Belfast, UK [61]. Refuge traps were positioned at the nodes of a 12×12 regular grid, with two meters between adjacent traps. The traps were examined at weekly intervals and the numbers of flatworms counted. Further details of the trapping procedure are provided in [38,61]. Here, we consider one record of trap counts made in January 2002 where, for the sake of comparison with the analysis of the preceding sections, we consider a sub-grid with 10 traps in each direction. The corresponding trap count data are shown in Figure 4a. The total number of flatworms collected from the sampling grid of N=100 locations is 454, which is a significantly higher number than the population abundance evaluated from trap counts on a similar grid for the slug populations. The flatworm spatial distribution visualized from the trap count data on a sampling grid of N=10×10=100 traps is shown in Figure 4b.

Whatever management action may be needed, the decision will only be well informed if it is based on a reliable, sufficiently accurate estimate of the average trap count. Therefore, evaluation of the average trap count still requires the condition of Equation (8) to hold. In order to make our analysis consistent with Section 3, we consider tolerance τ=0.25, i.e., the estimation error up to 25% is regarded as acceptable. We also introduce a hypothetical ‘management point’ $S_{T}$ of the average trap count, so that a management action can only be applied (e.g., aiming to ease the pressure on the earth worm population) if an estimate of the average trap count is larger than the given management point:

(9) $$S_{c}\geq S_{T}.$$

For the sake of discussion only, in our analysis below, we consider $S_{T}=5$.

We now apply the procedure described in Section 3 to the flatworm sampling data to coarsen the original fine sampling grid (cf. Figure 4a) and to investigate the statistical properties of the average trap count and of the estimation error calculated on various sub-grids. The results are shown in Table 3.

The results obtained for the flatworm average trap count on a sampling grid of N=25 locations show good accuracy as the upper bound $\mu_{e}\left(N\right)+\sigma_{e}\left(N\right)$ of the estimation error is 12%. Hence, the estimate of the average trap count can be taken as reliable. However, the comparison of the average trap count estimate to the management point $S_{T}$ is less straightforward. We recall from the previous section that the array of all possible values of the average trap count *S* can be quantified in at least two different ways, i.e., by calculating quantities $S_{soft}$ and $S_{hard}$. To increase the robustness of the estimation, both these quantities should be considered. For a coarse grid of N=25 sampling locations, $S_{hard}>S_{T}$, but $S_{soft}<S_{T}$. Whilst the former apparently recommends a management action, the latter does not. This ambiguity indicates that the ‘actual’, precise value of the average trap count (which is not available due to the insufficient information inherent for coarse grids) is very close to the management point. To make a decision whether a management action needs to be taken, one would have to use additional data.

Interestingly, the results obtained in the case of a coarser grid of N=9 locations appears to be easier to utilise. The ‘upper bound’ ($\mu_{e}+\sigma_{e}$) of the estimation error has now increased to 24.6%; however, since it is still below the required tolerance, the estimation can be regarded as reliable. In this case, both $S_{hard}>S_{T}$ and $S_{soft}>S_{T}$ consistently indicate that management action should be considered in spite of $\mu_{S}<S_{T}$.

In the cases of N=4 and N=1, $\bar{e}>\tau$, indicating that the accuracy of the population estimate, is unacceptably low. Hence, despite the finding that both $S_{hard}>S_{T}$ and $S_{soft}>S_{T}$, management action cannot be recommended: the true value of the average trap count is likely to be below the management point and an intervention is likely to be unjustified.

## 5. Analysis of Trap Count Data for the Carabid Beetle *Pterostichus melanarius*

**Figure 5:**
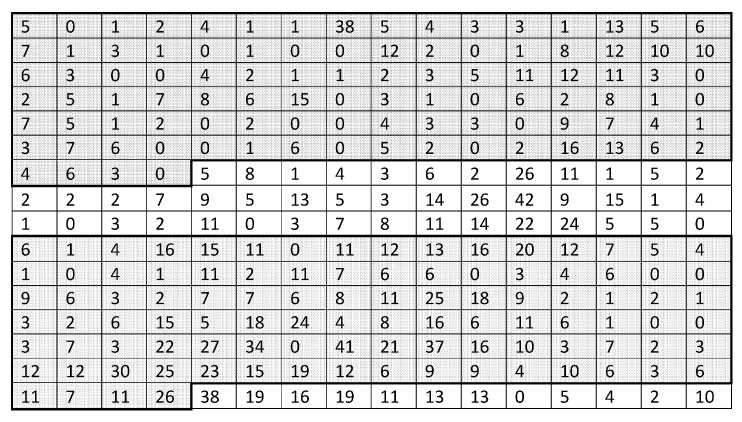
Two data sets of N=100 trap counts of *P. melanarius*; each have been extracted from a single original data set of N=256 trap counts collected by Alexander et al. (2005). Data Set *I* has been taken from the upper half of the original data table, and Data Set II has been taken from the lower half of the original data table. Both data sets are shadowed in grey.

The above conclusions on the accuracy of evaluation on coarse sampling grids are further corroborated by the analysis of the results of a field study on another species, the predatory carabid beetle *Pterostichus melanarius* [37]. Trap counts for this fast moving, very active species were obtained from a 16×16 regular grid of pitfall traps established in a winter wheat field in Devon, UK (Figure 5). Details of the trapping procedures are provided in [37]. For the purpose of the current study, two rectangular sub-grids, each with 100 sampling locations, were extracted from the original square grid of 16×16=256 traps. The two sub-grids are highlighted in grey in Figure 5. In our analysis below, we refer to the upper part of the original table as Data Set *I* and to the the lower part of the table as Data Set II.

*P. melanarius* is a beneficial species predating several pest species [36], so the concept of a control threshold is irrelevant. However, monitoring and management action may be needed for different reasons, e.g., in the context of conservation. One concern therefore is that the average trap count (considered as a proxy of the beetle’s population abundance) should not fall below a certain management point, say $S_{*}=2$ beetles per trap.

Before the average trap count can be compared with the management point, the randomness of population estimation on a coarse grid has to be considered. Therefore, as well as the above procedure (see Section 3), we calculate the array of values of the average trap count $S_{c}$ and the estimation error *e* on various sub-grids for Data Sets *I* and II. Once the statistical measures of the distribution of $S_{c}$ and *e* are established, one can consider two values that quantify the whole range of possible trap count values, namely, $S_{hard}$ and $S_{soft}$; see the lower two rows in Table 4 and Table 5. Note that, due to the different context—conservation rather than control—the definition of these values is different from Section 3 and Section 4). Whenever one of these values is below the management point, there is a concern; if both of them fall below the management point, then conservation measures become necessary.

First considering Data Set *I*, in order to make the results comparable with the analysis of the previous sections, before coarsening the grid we first rearrange the one hundred samples available in the data set as a 10×10 grid. The results of the coarse grid analysis are shown in Table 4. The total number of beetles on the baseline sampling grid of N=100 locations is 419 so that the average trap count S=4.19 (see the second column of the table). Since it is above the management point $S_{*}=2$, a management action is not required. Note that, because the bootstrapping procedure is not possible in this case, $S_{soft}=S_{hard}=S$.

The spatial distribution of the beetles in the corresponding area (see the top part of Figure 5) is mostly homogeneous with one relatively small patch with a high population density. However, the trap count taken from that patch contributes approximately 10% of all the beetles sampled, so may exert high leverage on the accuracy of evaluation. In particular, one can expect that a coarser grid that misses the patch would provide an estimate of unacceptable accuracy. This expectation is confirmed by the results shown in Table 4. Already for N=25, the upper bound of the estimation error is above the assumed value τ=0.25 of the tolerance and further increases on coarser sub-grids. Therefore, if the data are collected on a coarse sampling grid (N=25 or less), a reliable estimate of the average trap count is not possible, precluding a management decision. Even in a case where both $S_{soft}$ and $S_{hard}$ fall below the management point (e.g., see the N=4 column in Table 4), the ‘true’ average trap count is likely to be above the point, as the uncertainty of the estimate is very high. Interestingly, for all four coarse grids, i.e., for N=25, N=9, N=4, and N=1, the expected average trap count $\mu_{S}$ is almost the same, demonstrating that, even with the use of the bootstrapping procedure, a single statistical measure is not sufficient to provide a robust estimate.

In Data Set II, the total number of beetles recorded on the fine sampling grid of N=100 locations is 949, so that S=9.49. The average trap count is therefore well above the management point $S_{*}=2$; thus, no management action is needed. This value remains approximately the same on all coarse grids considered; see Table 5. Similarly to Data Set *I*, the standard deviations for the average count $\sigma_{S}$ and for the estimation error $\sigma_{e}$ increase with a decrease in *N*. However, the rate of increase is somewhat slower than in the case of Data Set *I*; as a result, for N=25 the upper bound of the estimation error is still below the tolerance. This is consistent with the properties of the beetle distribution over the corresponding area; see the bottom of Figure 5. Unlike Data Set *I*, there are three patches of high population density, not one. A distribution with multiple patches is known to be somewhat less sensitive to the number of sampling locations in the grid compared to the case of extreme population aggregation in one single patch [31,33,38].

Due to the large population counts in the case of Data Set II, both $S_{soft}$ and $S_{hard}$ are much higher than the hypothetical management point $S_{*}=2$ on the coarse grids with N=25, N=9, and N=4. In practical terms, this suggests that, even if the accuracy of estimation becomes low (e.g., 0.38 in case N=9), the true value of the average count *S* is unlikely to fall below $S_{*}$. We therefore conclude that the use of coarse sampling grids, in particular in the context of conservation, may still be justified for the population abundance evaluation when the average trap count is expected to be well above the management point.

## 6. Analysis of the Temporal Dynamics of Slug Populations

The conclusions on the use of coarse sampling grids for population abundance evaluation can be further tested by applying our approach to data describing temporal population dynamics where the average trap count may change significantly as time progresses. In this section, we consider the data describing slug abundance (see Section 2 for details) obtained from trap counts collected from the standard 10×10 grids established in two fields (Uppington and Stoney Lawn, Shropshire, UK) on several dates between December 2015 and May 2016. For each of the census dates (8 for Uppington; 12 for Stoney Lawn), the data were analysed using the procedure described in Section 3 to calculate the mean value $\mu_{S}$ of the average trap count and the standard deviation $\sigma_{S}$ on several sub-grids with a different number *N* of sampling locations. The range of possible values for the average trap count is then described by the interval $\left(\mu_{S}-\sigma_{S},\mu_{S}+\sigma_{S}\right)$.

**Figure 6:**
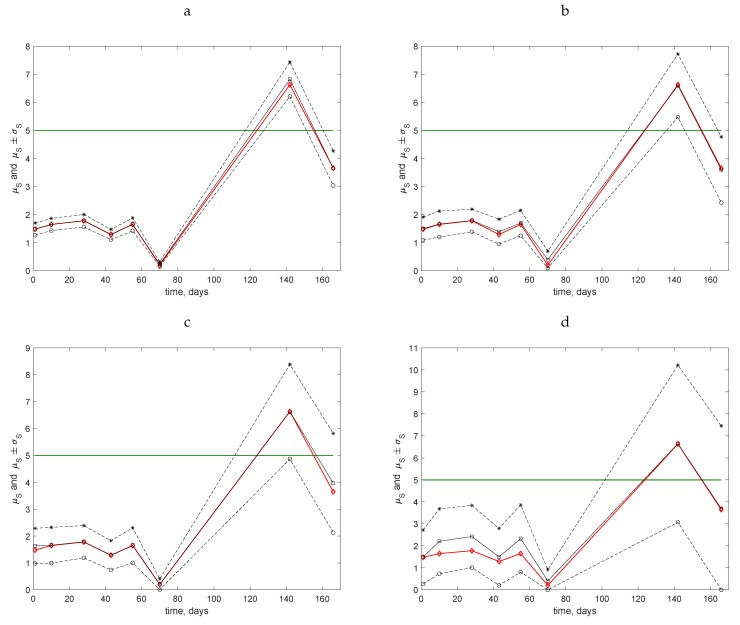
Temporal dynamics of slug populations in the Uppington field. Field assessments commenced on 7 December 2015 (Day 1). The mean value $\mu_{S}$ (solid black line, open squares), the ‘exact’ value *S* of the average trap count calculated on the original grid of N=100 locations (solid red line, open diamonds), the lower bound $\mu_{S}-\sigma_{S}$ (dashed line, open circles), and the upper bound $\mu_{S}+\sigma_{S}$ (dashed line, stars) of the range of the average trap count are shown. The hypothetical threshold value $S_{T}=5$ is shown by the horizontal solid green line. (**a**) Sampling grid of N=25 locations. (**b**) Sampling grid of N=9 locations. (**c**) Sampling grid of N=4 locations. (**d**) Sampling grid of N=1 location.

The results obtained for four coarse grids using the data from the Uppington field are shown in Figure 6: (a) for N=25, (b) for N=9, (c) for N=4, and (d) for N=1. In all of these cases, the mean value $\mu_{S}$ (solid black line, open squares) is presented along with the ‘exact’ value *S* of the average trap count (solid red line, open diamonds) calculated on the original grid of N=100 locations. The range $\mu_{S}\pm \sigma_{S}$ is shown by the dashed lines. The solid green line shows the hypothetical threshold value $S_{T}=5$.

For all dates during the first three months of observations, the average trap count was well below the threshold value $S_{T}$. Note that the value of $\mu_{S}$ calculated on coarse grids was always very close to the ‘exact’ average trap count, $\mu_{S}\approx S$, so that the range $\mu_{S}\pm \sigma_{S}$ is centred around the true value of the average count. However, a confident conclusion as to whether the population is below the threshold (above the threshold) can only be made if not only $\mu_{S}<S_{T}$ but also the entire range of possible values is below the threshold, i.e., $\mu_{S}+\sigma_{S}<S_{T}$ (respectively, $\mu_{S}-\sigma_{S}>S_{T}$). In other words, $S_{T}$ must lie outside of the interval $\left(\mu_{S}-\sigma_{S},\mu_{S}+\sigma_{S}\right)$. It can be readily seen that this unambiguous situation only occurs for grids with N=25 and N=9 locations: in Figure 6a,b, the horizontal line $S_{T}=5$ never intersects the range of average trap counts between the dashed lines for any of the census dates. Meanwhile, for the grids with N=4 (Figure 6c) and N=1 (Figure 6d), for the last two census dates, the value $S_{T}=5$ lies within the range of possible values of the average trap count so that no immediate conclusion can be made. In particular, for those two very coarse grids for the data collected on the last census date, the range of the average trap count is so large that an estimate $S_{c}<S_{T}=5$ can likely be obtained, while the true value (known from the fine grid) is actually $S>S_{T}$. We therefore conclude that the use of coarse grids may be justified (providing information of sufficient accuracy) when the average trap count is expected to lie well below the management point. However, it is hardly justified when the average trap count appears to be close to the management point.

**Figure 7:**
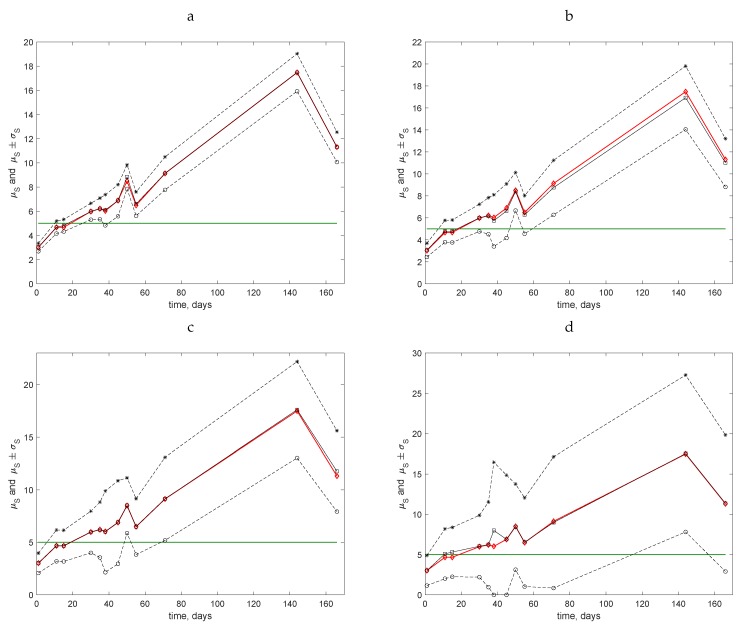
Temporal dynamics of slug populations in the Stoney Lawn field. Field assessments commenced on 7 December 2015 (Day 1). The mean value $\mu_{S}$ (solid black line, open squares), the ‘exact’ value *S* of the average trap count calculated on the original grid of N=100 locations (solid red line, open diamonds), the lower bound $\mu_{S}-\sigma_{S}$ (dashed line, open circles), and the upper bound $\mu_{S}+\sigma_{S}$ (dashed line, stars) of the range of the average trap count are shown. The hypothetical threshold value $S_{T}=5$ is shown by the horizontal solid green line. (**a**) Sampling grid of N=25 locations. (**b**) Sampling grid of N=9 locations. (**c**) Sampling grid of N=4 locations. (**d**) Sampling grid of N=1 location.

The data collected at the other, more densely populated field (Stoney Lawn) were analysed using a similar approach; see Figure 7. The situation differs from the previous case because, for all coarse grids, the line $S_{T}=5$ intersects the range $\mu_{S}\pm \sigma_{S}$ at least twice. Therefore, for several census dates, inaccurate estimates of the average trap count are likely.

In order to quantify the uncertainty arising in cases where the threshold $S_{T}$ lies within the interval $\left(\mu_{S}-\sigma_{S},\mu_{S}+\sigma_{S}\right)$, one can calculate the probability *p* of drawing a wrong conclusion:

(10) $$p=\frac{k}{K}$$

where *K* is the total number of realisations of the estimate $S_{c}$ of the average trap count *S* (emerging in the bootstrapping procedure, see Section 3) on a sampling grid of *N* locations, and *k* is the number of realisations for which values $S-S_{T}$ and $S_{c}-S_{T}$ have a different sign. If inaccurate estimates of the average trap count are likely to appear, then the probability *p* is high, while if accurate estimates of the average trap count appear on a rare occasion, then *p* is low.

The results of the probability computation on various sampling grids are shown in Table 6. It can be seen from the comparison of Table 6 and Figure 7 that the above conclusion is further confirmed: a fine sampling grid must be used for evaluation when the average trap count is close to the threshold value. Even on a relatively fine grid of N=25 sampling locations, we have p=0.28 on Day 11 of the measurements when the average trap count is S=4.67. Meanwhile, it also follows from the computation of the probability *p* that even a very coarse sampling grid can be used in the sampling routine if the average trap count is sufficiently far away from the threshold value.

## 7. Discussion and Concluding Remarks

Average trap counts have long been used to provide information on population abundance in ecological applications or when making management decisions [5,6,28]. Accurate and reliable evaluation of the average trap count is important when drawing conclusions about an agro-ecological problem. In this paper, to determine the impact of the number of sampling locations on the accuracy of an average trap count, the outcomes of assessments of three different invertebrate species were investigated using a series of coarse sampling grids. In all cases, it was concluded that the average trap count evaluated on coarse sampling grids should be considered as a random variable. The range of that variable is influenced by a number of factors that can be related to the biology and behaviour of the species sampled, such as the spatial pattern of the population distribution. Species such as the slug *D. reticulatum* display a heterogeneous spatial distribution, characterised by spatio-temporally stable patches of higher densities dispersed amongst areas of lower numbers [42,44]. In such situations, coarse sampling grids usually result in inaccurate evaluation of average trap count. In this paper, we have developed a method (see Section 3) that can provide an understanding of the degree of reliability of assessments made.

Given the commonly occurring limitations on available resources, the use of coarse grids of traps in routine monitoring of invertebrate populations is almost ubiquitous. The question therefore arises how the inherent uncertainty of the average trap count evaluation can be quantified and dealt with. It is demonstrated in this paper that the results of sampling on coarse grids of traps can only be considered as reliable if the mean value significantly differs from the management point, i.e., a species-specific target value. In this case, almost any realisation of a random average trap count is likely to be considerably different from the management point. However, in the case where the estimate of the average trap count is close to the management point, the sampling data collected on a coarse grid becomes insufficient. A management decision made based on the coarse grid data is then likely wrong or irrelevant. A larger sampling grid is required and the data needs to be analysed more carefully, e.g., using the modified bootstrapped procedure considered in Section 3.

As suggested in the introduction and shown in our previous work, it is the spatial heterogeneity (to which we somewhat loosely refer to as a spatial pattern) that makes the problem of the population abundance evaluation challenging. The importance of spatial pattern has been appreciated in many natural sciences, and a number of spatial-sensitive methods and approaches have been developed to account for the effects of spatial heterogeneity, e.g., geostatistical and point pattern methods [62,63]. However, a closer look at the existing approaches readily reveals that the problem of an accurate evaluation of the average sample count on coarse grids, which is central for many of those methods (e.g., the ordinary kriging method), has been poorly investigated. A clear understanding of this issue is lacking; instead, the problem is often circumvented by implicitly assuming that a good estimate of the average count should somehow be available.

The problem of evaluation of the average sample size is by no means new [20,22]. However, the majority of existing approaches are based on the assumption (sometimes implicit) that the sample size is described by a normal distribution. Only in this special case can the mean and the confidence interval of the estimation be readily obtained from a small number of samples (but see also the point below). Meanwhile, considerable evidence has been obtained over the last two decades to show that situations where an ecologically meaningful random variable is described by the normal distribution are rare [64,65]. The situation is exacerbated by the observation that, in many cases, random variables are described by distributions that do not have a finite variance (e.g., power laws), in which case the standard analysis becomes irrelevant, even when a very large number of samples is available. Another major assumption routinely made in the standard analysis is that the value of a single sample (or the mean value calculated based on a small number of samples) is in the centre of the confidence interval (cf. Lund et al. 1958, see especially the caption to Figure 2 in that paper). If this assumption is relaxed, the accuracy of the standard approach becomes problematic even in the case of normal distribution. In this paper, we have considered an approach that does not require the above assumptions.

It is perhaps not surprising that estimates based on a very small number of counts (ultimately, just one count) are unreliable. An interesting aspect of our study is how intermediate sampling efforts can provide reliable estimates. Our approach offers a means of controlling accuracy if a moderately coarse grid is used in the monitoring protocol. Given the results of sampling, further coarsening of a sampling grid can be applied, similar to that carried out in Section 3. This also allows for assessment of the accuracy of the original ‘fine’ grid. By comparing the results obtained on the series of coarsened grids (see Table 1, Table 2, Table 3 and Table 4), the evaluation error shows a clear monotone increase with grid coarsening. For instance, Table 1 shows that $\sigma_{s}$ roughly doubles at each step when N decreases from 25 to 9 to 4 to 1. It seems reasonable to expect that this tendency can be extended to the full grid; correspondingly, $\sigma_{s}$ should decrease about twofold when the full grid of 100 samples is decreased to 25 samples. It is then readily seen that the accuracy of the fine grids used in Section 3, Section 4 and Section 5 is below the suggested tolerance of 25%. However, should the mean values of the average trap count obtained on coarsened sub-grids appear to be very different from the average trap count on the original grid, and the range of possible average trap count values be large, then the results obtained on the original grid would not be reliable.

As is demonstrated in this paper, our approach can be applied to a broad range of invertebrate species with widely differing biological and behavioural characteristics. For example, in arable fields *D. reticulatum*, an indigenous mollusc is characterised by a highly heterogeneous spatial distribution [43] with the stability of the relatively discrete patches of higher population densities being enhanced by the species’ slow dispersal rate and responses to environmental factors. The New Zealand flatworm (*A. triangulatus*) is an invasive free living planarian predator feeding on earthworms. At the edges of its geographical range, it can be restricted to refuges offering micro-habitats with favourable conditions for its survival, again resulting in a heterogeneous population distribution [54]. Nearer to the centre of its geographical range, however, a more homogeneous distribution can be found in favourable habitats. In such cases, the approach investigated in this study offers a method of modifying sampling strategy to optimise assessment of average trap catches (average population density), thus allowing more accurate ecological interpretation and comparisons between different parts of its range. The carabid beetle *P. melanarius* also displays very different biological and behavioural characteristics, being an active insect predator that is known to range widely within and between arable fields. Patterns of population redistribution in the field can be explained by complex movement behaviour both in crops and at the habitat interface, making accurate interpretation of average trap catches essential when studying such populations [66]. Similarly, the spatial distribution of the development and reproduction of the same species [67] will also affect the heterogeneity of sampled populations, adding to the complexity of the problem. The technique investigated in this study may be used to ensure more accurate and comparable population assessments to be conducted despite the complex distribution/dispersion patterns of such species resulting from their biology.

In this paper, we have assumed that, at any given sampling location, the trap count is a reliable proxy of the actual animal abundance in the vicinity of the trap. It should be mentioned that calculating the population density from the trap count value(s) is not straightforward as it can be affected by a variety of factors, in particular, by animals’ movement behaviour [14,15,16,68]. A general observation—often referred to as the ‘activity-density paradigm’ [69] —is that the same trap count may result from a scarce population of fast moving animals and from a less dense population of slower moving animals. Therefore, trap counts have a relative rather than absolute meaning. A detailed consideration of these issues lies beyond the scope of this paper.

Note that the goal of this paper is to discuss the problem of coarse grid sampling in the contexts of conservation and pest control (i.e., where a comparison of the average trap count to the management point may be required) rather than to provide a method fully ready for practical applications in the field. In particular, a practical method should include a full understanding of the effect of various spatial arrangements of samples (e.g., regular, random, transect, etc.) and to give recommendations accordingly. Although our analysis suggests that the exact spatial arrangements of the traps may be less important that their number (as follows from the bootstrapping results), this issue requires a more careful, detailed analysis. Such analysis should become a focus of future work.

## Figures and Tables

**Table 1:** Evaluation of the average trap count on coarse sampling grids. Analysis of the evaluation accuracy for the slug distribution from data taken in the Oadby field on 02 September 2016. The mean value $\mu_{S}\left(N\right)$ and the standard deviation $\sigma_{S}\left(N\right)$ are computed for the average trap count $S_{c}$ on a sequence of quasi-regular grids with number *N* of sampling locations. For any realisation of the average trap count the error (2) is computed. Given *K* realisations of error (2), the mean evaluation error $\mu_{e}\left(N\right)$, the standard deviation $\sigma_{e}\left(N\right)$ of the evaluation error, and the upper error bound $\mu_{e}\left(N\right)+\sigma_{e}\left(N\right)$ are computed for each *N* in the table. The values of the estimate of the average trap count that exceed a hypothetical management action threshold of 1.5 slugs per trap (in this case, suggesting application of control measures) are shown in red.

*N*	100	25	9	4	1
$\mu_{S}\left(N\right)$	1.07	1.09205	1.10693	1.06380	1.09375
$\sigma_{S}\left(N\right)$	0.0	0.191571	0.349385	0.53295	1.04197
$\mu_{e}\left(N\right)$	0.0	0.137604	0.26325	0.401503	0.800444
$\sigma_{e}\left(N\right)$	0.0	0.100688	0.192173	0.287855	0.638311
$\bar{e}=\mu_{e}\left(N\right)+\sigma_{e}\left(N\right)$	0.0	0.238292	0.455424	0.689358	1.43876
$S_{soft}=\mu_{S}+\sigma_{S}$	1.07	1.283621	1.456315	**1.59675**	**2.13572**
$S_{hard}=\mu_{S}\left(1+\bar{e}\right)$	1.07	1.352276	**1.611052**	**1.797139**	**2.667393**

**Table 2:** Evaluation of the average trap count on coarse sampling grids. Analysis of the evaluation accuracy for the slug distribution from data taken in various agricultural fields (as shown in the table). The mean value $\mu_{S}\left(N\right)$ and the standard deviation $\sigma_{S}\left(N\right)$ are computed for the average trap count $S_{c}$ on a sequence of quasi-regular grids with number *N* of sampling locations. For any realisation of the average trap count the error (2) is computed. Given *K* realisations of error (2), the mean evaluation error $\mu_{e}\left(N\right)$, the standard deviation $\sigma_{e}\left(N\right)$ of the evaluation error, and the upper error bound $\mu_{e}\left(N\right)+\sigma_{e}\left(N\right)$ are computed for each *N* in the table.

**Field: *Adeney Middle, 05.09.2016***					
N	**100**	**25**	**9**	**4**	**1**
$\mu_{S}\left(N\right)$	0.58	0.580306	0.574507	0.659841	0.601875
$\sigma_{S}\left(N\right)$	0.0	0.13028	0.254346	0.402294	0.75277
$\mu_{e}\left(N\right)$	0.0	0.180296	0.348967	0.543847	1.15625
$\sigma_{e}\left(N\right)$	0.0	0.121942	0.251273	0.371136	0.69694
$\mu_{e}\left(N\right)+\sigma_{e}\left(N\right)$	0.0	0.302238	0.60024	0.914983	1.85319
**Field:** ***South Kyme MC, 25.11.2016***					
N	**100**	**25**	**9**	**4**	**1**
$\mu_{S}\left(N\right)$	1.12	1.12206	1.18208	1.19131	1.40625
$\sigma_{S}\left(N\right)$	0.0	0.228638	0.426481	0.679341	1.40987
$\mu_{e}\left(N\right)$	0.0	0.168173	0.315422	0.480558	0.94519
$\sigma_{e}\left(N\right)$	0.0	0.121077	0.239031	0.330425	0.73124
$\mu_{e}\left(N\right)+\sigma_{e}\left(N\right)$	0.0	0.28925	0.554353	0.810983	1.67643
**Field:** ***Uppington, 13.09.2016***					
N	**100**	**25**	**9**	**4**	**1**
$\mu_{S}\left(N\right)$	0.5	0.499703	0.549732	0.487895	0.55
$\sigma_{S}\left(N\right)$	0.0	0.129847	0.257785	0.397644	1.17853
$\mu_{e}\left(N\right)$	0.0	0.218399	0.442847	0.633147	1.77875
$\sigma_{e}\left(N\right)$	0.0	0.151841	0.311785	0.523172	1.17853
$\mu_{e}\left(N\right)+\sigma_{e}\left(N\right)$	0.0	0.37024	0.754633	1.15632	2.95728

**Table 3:** Results for the flatworm average trap counts obtained on a sequence of coarse grids from the data in Figure 4a. The mean value $\mu_{S}\left(N\right)$ and the standard deviation $\sigma_{S}\left(N\right)$ are computed for the average trap count $S_{c}$ on a sequence of quasi-regular grids with number *N* of sampling locations. For any realisation of the average trap count the error (2) is computed. Given *K* realisations of error (2), the mean evaluation error $\mu_{e}\left(N\right)$, the standard deviation $\sigma_{e}\left(N\right)$ of the evaluation error, and the upper error bound $\mu_{e}\left(N\right)+\sigma_{e}\left(N\right)$ are computed for each *N* in the table. The values of the estimate of the average trap count that exceed the hypothetical management point of $S_{T}=5$ flatworms per trap are shown in red.

*N*	100	25	9	4	1
$\mu_{S}\left(N\right)$	4.54	4.53945	4.81888	4.5333	5.25875
$\sigma_{S}\left(N\right)$	0.0	0.391353	1.01255	1.29929	3.17851
$\mu_{e}\left(N\right)$	0.0	0.069316	0.142631	0.229832	0.573995
$\sigma_{e}\left(N\right)$	0.0	0.0511691	0.103361	0.166017	0.368445
$\bar{e}=\mu_{e}\left(N\right)+\sigma_{e}\left(N\right)$	0.0	0.120561	0.245992	0.395849	0.942441
$S_{soft}=\mu_{S}+\sigma_{S}$	4.54	4.931353	**5.83143**	**5.83259**	**8.43726**
$S_{hard}=\mu_{S}\left(1+\bar{e}\right)$	4.54	**5.086731**	**6.004286**	**6.327802**	**10.21481**

**Table 4:** Results for the average trap counts obtained for the beetle population on a sequence of coarse grids from Data Set *I* in Figure 5. The mean value $\mu_{S}\left(N\right)$ and the standard deviation $\sigma_{S}\left(N\right)$ are computed for the average trap count $S_{c}$ on a sequence of quasi-regular grids with number *N* of sampling locations. For any realisation of the average trap count the error (2) is computed. Given *K* realisations of error (2), the mean evaluation error $\mu_{e}\left(N\right)$, the standard deviation $\sigma_{e}\left(N\right)$ of the evaluation error, and the upper error bound $\mu_{e}\left(N\right)+\sigma_{e}\left(N\right)$ are computed for each *N* in the table. The estimated values of the average trap count that falls below the hypothetical management point (conservation target) of $S_{*}=2$ beetles per trap are shown in red.

*N*	100	25	9	4	1
$\mu_{S}\left(N\right)$	4.19	4.19118	4.22304	4.20802	4.26312
$\sigma_{S}\left(N\right)$	0.0	0.831115	1.59125	2.54429	5.0996
$\mu_{e}\left(N\right)$	0.0	0.160162	0.291408	0.462822	0.800091
$\sigma_{e}\left(N\right)$	0.0	0.117191	0.241096	0.425638	0.912036
$\bar{e}=\mu_{e}\left(N\right)+\sigma_{e}\left(N\right)$	0.0	0.277352	0.532503	0.888461	1.71213
$S_{hard}=\mu_{S}-\sigma_{S}$	4.19	3.360065	2.63179	**1.66373**	**<0**
$S_{soft}=\mu_{S}\left(1-\bar{e}\right)$	4.19	3.028748	**1.974258**	**0.469358**	**<0**

**Table 5:** Results for the average trap counts obtained for the beetle population on a sequence of coarse grids from Data Set II in Figure 5. The mean value $\mu_{S}\left(N\right)$ and the standard deviation $\sigma_{S}\left(N\right)$ are computed for the average trap count $S_{c}$ on a sequence of quasi-regular grids with number *N* of sampling locations. For any realisation of the average trap count the error (2) is computed. Given *K* realisations of error (2), the mean evaluation error $\mu_{e}\left(N\right)$, the standard deviation $\sigma_{e}\left(N\right)$ of the evaluation error, and the upper error bound $\mu_{e}\left(N\right)+\sigma_{e}\left(N\right)$ are computed for each *N* in the table. The estimated values of the average trap count that falls below the hypothetical management point (conservation target) of $S_{*}=2$ beetles per trap are shown in red.

*N*	100	25	9	4	1
$\mu_{S}\left(N\right)$	9.49	9.48836	9.32295	9.47697	9.54531
$\sigma_{S}\left(N\right)$	0.0	1.53983	2.65411	3.99768	8.54964
$\mu_{e}\left(N\right)$	0.0	0.129577	0.225206	0.33965	0.725803
$\sigma_{e}\left(N\right)$	0.0	0.0964533	0.164412	0.247537	0.56588
$\bar{e}=\mu_{e}\left(N\right)+\sigma_{e}\left(N\right)$	0.0	0.22633	0.389618	0.587188	1.29168
$S_{hard}=\mu_{S}-\sigma_{S}$	9.49	7.94853	6.66884	5.47929	**0.99567**
$S_{soft}=\mu_{S}\left(1-\bar{e}\right)$	9.49	7.340859	5.690561	3.912207	**<0**

**Table 6:** The probability of drawing a wrong conclusion about the slug population in the Stoney Lawn field. *N* is the number of sampling locations.

***N* = 25**												
day	1	11	15	30	35	38	45	50	55	71	144	166
*p*	0.0	0.27	0.26	0.06	0.07	0.21	0.07	0.0	0.05	0.0001	0.0	0.0
***N* = 9**												
day	1	11	15	30	35	38	45	50	55	71	144	166
*p*	0.002	0.35	0.36	0.21	0.25	0.42	0.27	0.015	0.25	0.036	0.0	0.0
***N* = 4**												
day	1	11	15	30	35	38	45	50	55	71	144	166
*p*	0.03	0.42	0.42	0.33	0.36	0.46	0.37	0.06	0.30	0.10	0.0	0.005
***N* = 1**												
day	1	11	15	30	35	38	45	50	55	71	144	166
*p*	0.0	0.44	0.49	0.41	0.5	0.62	0.52	0.22	0.46	0.33	0.04	0.13
